# SEED: a tool for disseminating systematic review data into Wikipedia

**DOI:** 10.1186/s13643-017-0607-3

**Published:** 2017-10-17

**Authors:** Lena Schmidt, Johannes Friedel, Clive E. Adams

**Affiliations:** 1 Hochschule Furtwangen University of Applied Sciences in Furtwangen im Schwarzwald, Furtwangen im Schwarzwald, Germany; 2 University of Nottingham, Nottingham, UK; 3 Institute of Mental Health, Jubilee Campus, University of Nottingham Innovation Park, Triumph Road, Nottingham, NG7 2TU UK

**Keywords:** Wikipedia, Summary of findings, Automation, Systematic reviews, Reducing waste

## Abstract

Wikipedia, the free-content online encyclopaedia, contains many heavily accessed pages relating to healthcare. Cochrane systematic reviews contain much high-grade evidence but dissemination into Wikipedia has been slow. New skills are needed to both translate and relocate data from Cochrane reviews to implant into Wikipedia pages. This letter introduces a programme to greatly simplify the process of disseminating the summary of findings of Cochrane reviews into Wikipedia pages.

## Background

Wikipedia contains over 40 million articles with 5.3 m in the English language [1]. Since its creation in 2001, Wikipedia has expanded to attract over 374 million unique visitors each month and around 20% of healthcare-related online searches direct to Wikipedia pages [2, 3]. Every year health pages on Wikipedia receive over 4.8 billion views [4]. Wikipedia is openly editable so any user can access and edit the majority of articles. Wikipedia policy states, however, that all information presented in pages must be “verifiable against a published reliable source” [5].

The Cochrane Collaboration is a non-for profit organisation producing, and maintaining systematic reviews of health care [6]. A systematic review “attempts to collate all empirical evidence that fits pre-specified eligibility criteria in order to answer a specific research question. It uses explicit, systematic methods that are selected with a view to minimising bias, thus providing more reliable findings from which conclusions can be drawn and decisions made” [7]. These findings are acceptable on Wikipedia pages if referenced and reliable. The Cochrane Collaboration uses a writing tool—RevMan [8]—to produce its reviews but disseminating the findings of the reviews within Wikipedia necessitates more work. Data have to be extracted, summarised and referenced in the clear and simple way required by Wikipedia. This additional effort and skill set often results in Cochrane reviews being less used in Wikipedia pages than could be the case.

## Aim

To produce a tool (Systematic EvidEnce Disseminator, SEED) to auto-generate a Wikipedia-compatible table and accompanying reference direct from Cochrane’s RevMan files.

## Procedure

The programme was created by students of Applied Health Science with close-to-zero previous knowledge in programming and the help of printed popular texts [9], numerous YouTube tutorials and internet fora. For creating the SEED tool, the integrated development environment ‘Eclipse’ was used (Neon version) [9]. This open-source programme allows the developed application to run on the programming language Java.

RevMan (v5.3) is an open-access text editor for authors of reviews [8]. It produces structured XML (Extensible Markup Language) review files (.rm5) within which Summary of Findings tables (SoF)—in turn produced by GradePro [10]—are embedded. Because the development team worked within Cochrane Schizophrenia’s editorial base they had access to RevMan files. The team had identified Wikipedia pages specific enough to be appropriately seeded with evidence from particular reviews’ SoF tables [11].

Because each .rm5 file has basically the same structure, this makes it simple to read content automatically. Eclipse parses the XML structure with its ‘chapters’ and ‘subchapters’ (children). The parser programme reads and follows relevant roots of XML-format and can jump to the SoF tables section and the references. In combination with the code we generated to parse through the files attributes or elements of the SoF part of the XML file can be converted into ‘strings’ and these, in turn, embedded in prepared phrases. These phrases were developed with the help of Sense about Science [12], to ensure both accuracy and clarity for Wikipedia readers. After SEED captures the SoF content, it converts it into the special format of Wikipedia tables. This format is standardised [13] and includes code for generating the correct size, font, formatting, colour and shading. We used Swing [14] to build the graphical interface of SEED. Swing is a GUI (Graphical user interface) tool allowing those with low IT knowledge to create a typical window with buttons, text fields and icons.

## Output

**Fig. 1:**
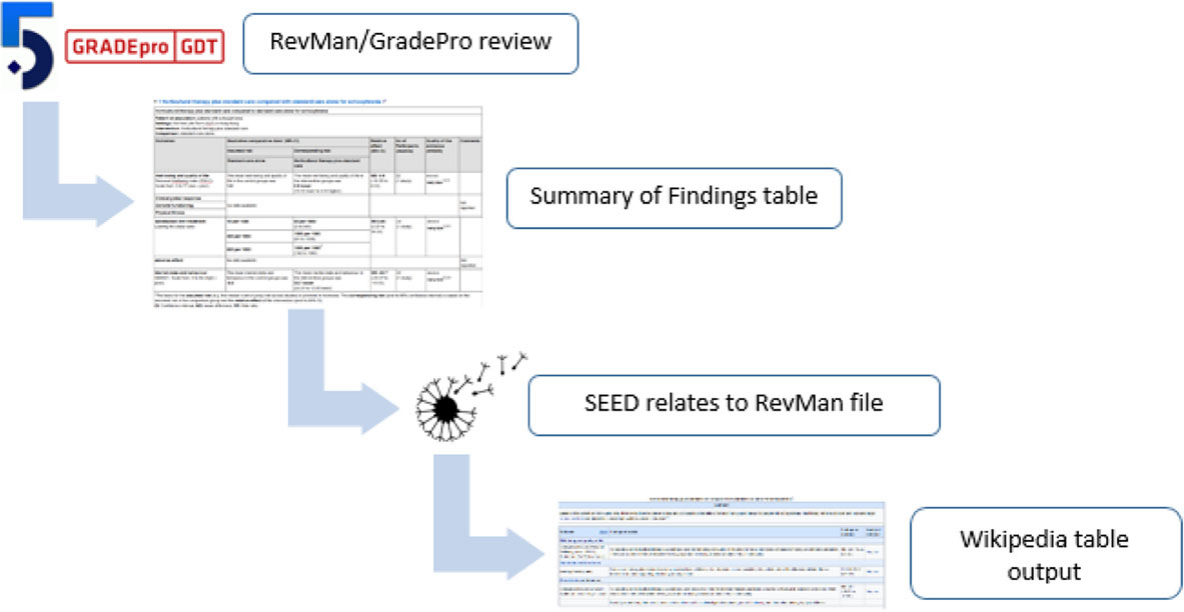
Information flow diagram

**Fig. 2:**
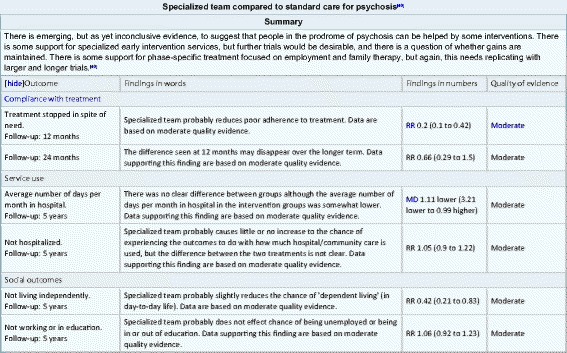
Output of SEED as it appears in Wikipedia

SEED allows the user to select their RevMan file, choose the relevant SoF table, and then produce compatible Code of Wikipedia tables (Fig. 1) that can be pasted directly into the editing box of the relevant Wikipedia page. This code will produce a clear and attractive table with an accompanying reference to that Cochrane review. One such ‘effect of treatment’ table is embedded in the Wikipedia page relevant to Early Intervention in Psychosis—Fig. 2 [15].

## Conclusions

The time of researchers and staff involved in knowledge transfer is finite. As the reviewing process becomes more sophisticated and dissemination in one format is increasingly seen as inadequate more shortcuts will be needed to produce output. SEED is open-access (see Availability of data and materials, below) and greatly increases the efficiency of knowledge transfer into one highly-accessed format.

## Abbreviations

EU: European Union
GUI: Graphical user interface
NIHR: National Institute of Health Research
SEED: Systematic EvidEnce Disseminator
SoF: Summary of Findings tables
XML: Extensible Markup Language
